# Anomalous current–voltage characteristics of SFIFS Josephson junctions with weak ferromagnetic interlayers

**DOI:** 10.3762/bjnano.11.19

**Published:** 2020-01-23

**Authors:** Tairzhan Karabassov, Anastasia V Guravova, Aleksei Yu Kuzin, Elena A Kazakova, Shiro Kawabata, Boris G Lvov, Andrey S Vasenko

**Affiliations:** 1 National Research University Higher School of Economics, 101000 Moscow, Russia; 2 Skolkovo Institute of Science and Technology, 121205 Moscow, Russia; 3 Department of Physics, Moscow State Pedagogical University, 119992 Moscow, Russia; 4 Sechenov First Moscow State Medical University, 119991 Moscow, Russia; 5 National Institute of Advanced Industrial Science and Technology,1-1-1 Umezono, Tsukuba, Ibaraki 305-8563, Japan; 6 I.E. Tamm Department of Theoretical Physics, P.N. Lebedev Physical Institute, Russian Academy of Sciences, 119991 Moscow, Russia

**Keywords:** current–voltage characteristics, Josephson junctions, proximity effect, superconductivity, superconductor/ferromagnet hybrid nanostructures

## Abstract

We present a quantitative study of the current–voltage characteristics (CVC) of SFIFS Josephson junctions (S = bulk superconductor, F = metallic ferromagnet, I = insulating barrier) with weak ferromagnetic interlayers in the diffusive limit. The problem is solved in the framework of the nonlinear Usadel equations. We consider the case of a strong tunnel barrier such that the left SF and the right FS bilayers are decoupled. We calculate the density of states (DOS) in SF bilayers using a self-consistent numerical method. Then we obtain the CVC of corresponding SFIFS junctions, and discuss their properties for different set of parameters including the thicknesses of ferromagnetic layers, the exchange field, and the magnetic scattering time. We observe an anomalous nonmonotonic CVC in case of weak ferromagnetic interlayers, which we attribute to DOS energy dependencies in the case of small exchange fields in the F layers.

## Introduction

It is well known that superconductivity and ferromagnetism are two competing antagonistic orders. In superconductors (S) electrons form Cooper pairs with opposite spins and momenta, while in ferromagnetic metals (F) electron spins tend to align in parallel. Nevertheless, it is possible to combine S and F layers in one hybrid structure, which leads to the observation of many striking phenomena. The reason is the superconducting proximity effect, i.e., the superconducting correlations leakage into a ferromagnetic metal due to Andreev reflection [1–7]. As a consequence, the real part of the pair wave function exhibits damped oscillatory behavior in a ferromagnetic metal. Hence, since the oscillations are spatially dependent, it is possible to realize a transition from “0” to “π” phase states in S/F/S structures upon changing the F layer thickness [1]. The proximity effect is characterized by the two length scales of decay and oscillations of the real part of the pair wave function in a ferromagnetic layer, ξ_f1_ and ξ_f2_, correspondingly [1]. If we consider the exchange field *h* as the only important parameter of a ferromagnetic material, both lengths are equal to 
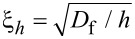
, where *D*_f_ is the diffusion constant in the ferromagnetic metal.

The existence of such phenomena enables the creation of so-called Josephson π junctions with a negative critical current [1–2]. Oscillations of the pair wave function in the F layer leads to several interesting phenomena in S/F/(S) systems, including nonmonotonic critical temperature dependence [8–12], Josephson critical current oscillations [13–41], and density of states (DOS) oscillations [42–45]. S/F hybrid structures have many promising applications in, e.g., single-flux quantum circuits [46–47], spintronic devices [48], memory elements [49–58] and spin-valves [59–65], magnetoelectronics [66–68], qubits [69], artificial neural networks [70], microrefrigerators [71–72], and low-temperature sensitive electron thermometers [73].

However, junctions with a ferromagnetic interlayer as well as other normal metal junctions (for example, SFNFS), proposed as elements of novel superconducting nanoelectronics, have limited applicability since such junctions have low resistance values [74–75]. This situation is resolved by addition of an insulating barrier (I) yielding a SFIFS layer sequence, which allows one to realize much larger values of the product *I*_c_*R*_n_, where *I*_c_ is the critical current of the junction and *R*_n_ its normal state resistance [36–38]. Recently, SIFS junctions attracted much attention and have been extensively studied both experimentally [32–41] and theoretically [23,45,76–80]. For instance, the current–voltage characteristics (CVC) of SIFS Josephson junctions with a strong insulating layer were studied in [45]. They exhibit interesting nonmonotonic behavior for weak ferromagnetic interlayers, i.e., small enough exchange fields. The reason for this behavior is the shape of the density of states in the F layer. At small exchange fields the decay length of superconducting correlations in the ferromagnetic material, ξ*_h_*, is large enough, which leads to profound variations of the superconducting density of states in the F layer as a function of the energy and results in a corresponding CVC behavior. With an increase of the exchange field the ξ*_h_* decreases, which suppresses the superconducting correlations in the F layer and makes the SIFS CVC similar to the *I*–*V* curve of the FIS junction.

**Figure 1:**
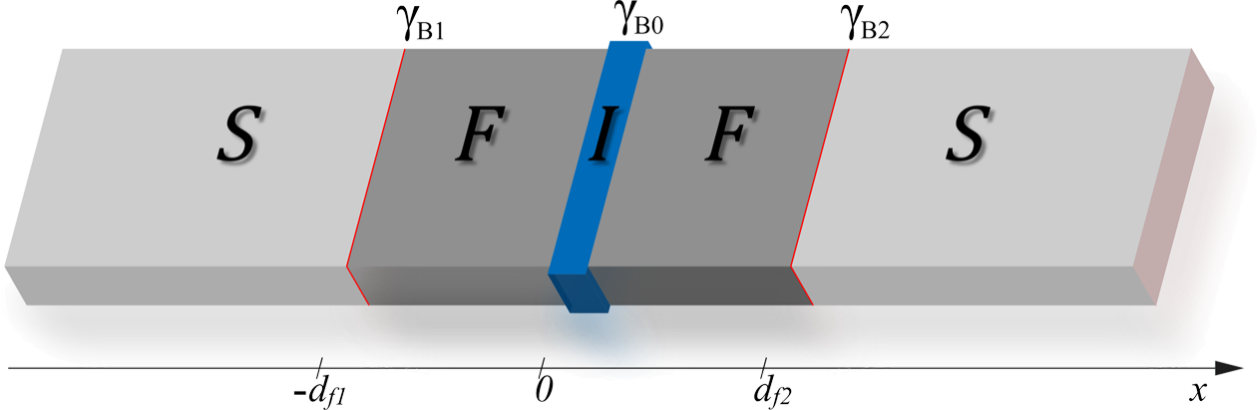
Schematic representation of the SFIFS hybrid structure (here S is a superconductor, F is a ferromagnetic metal and I is an insulating barrier). The thicknesses of the ferromagnetic interlayers are *d*_f1_ and *d*_f2_, correspondingly. The transparency of the left S/F interface is characterized by the parameter γ_B1_, while the transparency of the right F/S interface is characterized by the parameter γ_B2_. Both parameters γ_B1_, γ_B2_ ≪ 1, which corresponds to transparent metallic interfaces. The insulating barrier between the left and right interfaces (I) is described by γ_B0_ ≫ 1.

In this paper we study the current–voltage characteristics of SFIFS Josephson junctions with two ferromagnetic interlayers. SFIFS structures were also proposed for various applications in memory elements [56–58], single-flux quantum circuits [47], and as injectors in superconductor–ferromagnetic transistors [81–84], which can be used as amplifiers for memory, digital, and RF applications. In this work we study the current–voltage characteristics of a SFIFS junction as shown below in Figure 1. We present a quantitative model of the quasiparticle current in SFIFS junctions for different sets of parameters characterizing the ferromagnetic interlayers. In case of weak ferromagnetic metals we find an anomalous nonmonotonic shape of the current–voltage characteristics at subgap voltages and compare the results with the CVC of SIFS junctions [45]. We ascribe this behavior to DOS energy dependencies in case of small exchange fields in the F layers. The shape is smeared if we include a finite magnetic scattering rate. The anomalous nonmonotonic shape of the current–voltage characteristics of SFIFS junctions with weak ferromagnetic layers looks similar to the fine structures of quasiparticle currents, recently obtained experimentally on similar systems [82–85].

The paper is organized as follows. In the first section (“Model”) we formulate the theoretical model and basic equations and introduce the self-consistent numerical iterative method for calculating the density of states in S/F bilayers. In the next section (“Results and Discussion”) we present and discuss the results for the density of states in S/F bilayers in case of subgap values of the exchange field and the current–voltage characteristics of SFIFS junctions. Finally we summarize the results in the last section (“Conclusion”).

## Model

In this section we present the theoretical model we use in our studies. The geometry of the considered system is depicted in Figure 1. It consists of two superconducting electrodes and a pair of ferromagnetic interlayers, with thicknesses *d*_f1_ and *d*_f2_. The system contains three interfaces: two S/F (superconductor/ferromagnet) boundaries and one tunnel F-I-F interface. Each of these interfaces is described by the dimensionless parameter γ_Bj_ = *R*_Bj_σ*_n_*/ξ*_n_* (*j* = 0, 1, 2), which is proportional to the resistance *R*_Bj_ across the interface [86–88]. Here σ*_n_* is the conductivity of the F layer and 
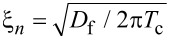
 is the coherence length, where *T*_c_ is the critical temperature of the superconductor S (here and below we assume ℏ = *k*_B_ = 1). In this paper we consider the diffusive limit, when the elastic scattering length 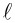 is much smaller than the characteristic decay length of the real part of the pair wave function in the ferromagnet, ξ_f1_, which we introduce later in Equation 1 and Equation 2. We assume that the S/F interfaces are not magnetically active. We also neglect the nonequilibrium effects [89–91] and use the Matsubara Green’s functions technique, which has been developed to describe many-body systems in equilibrium at finite temperature [92].

In our model the tunneling barrier is located between two F layers at *x* = 0 (Figure 1), whereas the other interfaces at *x* = *−d*_f1_ and *x* = *d*_f2_ are identical and transparent. This case corresponds to γ_B1_ = γ_B2_ ≪ 1 and γ_B0_ ≫ 1. In case of a sufficiently strong tunnel barrier (γ_B0_ ≫ 1), the two S/F bilayers in the SFIFS junction are decoupled, i.e., the amplitudes of two-electron processes between left and right F layers are negligibly small. Hence, the quasiparticle current through the SFIFS junction, biased by the voltage *eV*, can be calculated by using the Werthamer formula [93],

[3]
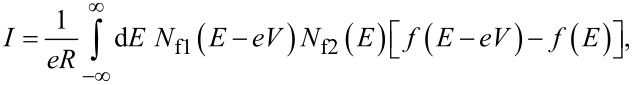


where *N*_f1,2_(*E*) are the densities of states (DOS) in the corresponding ferromagnetic layer at *x* = 0, *f*(*E*)= [1 + *e^E/T^*]^−1^ is the Fermi–Dirac distribution function, and *R* = *R*_B0_ is the resistance across the F-I-F interface. Both densities of states *N*_f1,2_(*E*) are normalized to their values in the normal state.

In order to obtain the densities of states in ferromagnetic layers, *N*_f1,2_(*E*), we use a self-consistent two-step iterative procedure, described below. As far as γ_B0_ ≫ 1, we can neglect the influence of the right F layer on the density of states in the left S/F bilayer and vice versa (see Figure 1). Thus we need to obtain the DOS at the outer border of each S/F bilayer. That can be done by solving the Usadel equations in the S/F bilayer system [94].

In the following, we use the θ-parameterizations of normal (*G* = cos θ) and anomalous (*F* = sin θ) Green’s functions and write the Usadel equations in the F layers in the form [94–95],

[4]
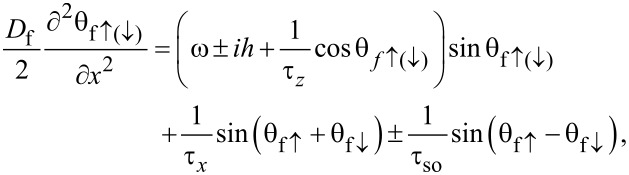


where the positive and negative signs correspond to the spin-up (“↑”) and spin-down (“↓”) states, respectively. In terms of the electron fermionic operators ψ_↑(↓)_ the spin-up state corresponds to the anomalous Green’s function *F*_↑_ ∼ ⟨ ψ_↑_ψ_↓_⟩, while spin-down state corresponds to *F*_↓_ ∼ ⟨ψ_↓_ψ_↑_⟩. The expressions ω = 2π*T*(*n* + 1/2) are the Matsubara frequencies, where *n* = 0, ±1, ±2, …, and *h* is the exchange field in the ferromagnet. The scattering times are labeled here as τ*_z_*, τ*_x_*, and τ_so_, where τ*_z(x)_* corresponds to the magnetic scattering parallel (perpendicular) to the quantization axis, and τ_so_ is the spin–orbit scattering time [96–99].

Assuming a strong uniaxial anisotropy in ferromagnetic materials, in which case there is no coupling between spin-up and spin-down electron populations, we neglect τ*_x_* (τ*_x_*^−1^ ≈ 0). We also assume the ferromagnets to have a weak spin–orbit coupling and thus neglect the spin–orbit scattering time τ_so_. After taking into account all the assumptions, the Usadel equations in the ferromagnetic layers for different spin states can be written as

[5]
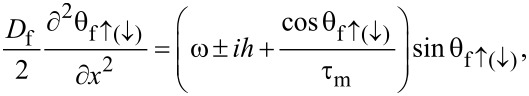


where τ_m_ ≡ τ*_z_* is the magnetic scattering time. In the superconducting layer S the Usadel equation reads [94]

[6]
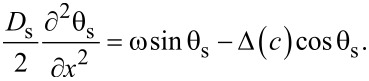


Here *D*_s_ is the diffusion coefficient in the S layer and Δ(*x*) is the pair potential in the superconductor. We note that Δ(*x*) vanishes in the F layer.

Equation 5 and Equation 6 must be supplemented with corresponding boundary conditions. At the S/F interfaces we apply the Kupriyanov–Lukichev boundary conditions. For example, at the left S/F interface they are written as [86],

[7]
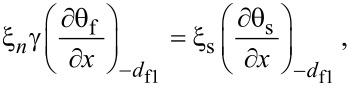


[8]
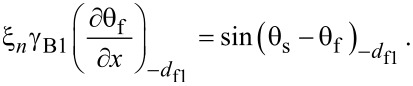


Similar equations can be written at the right S/F interface at *x* = *d*_f2_. Here γ = ξ_s_σ*_n_*/ξ*_n_*σ_s_, where σ_s_ is the conductivity of the S layer and 
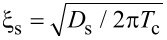
 is the superconducting coherence length. The parameter γ defines the strength of the inverse proximity effect, i.e., the suppression of superconductivity in the adjacent S layer by the ferromagnetic layer F. We consider the parameter γ to be relatively small γ ≪ 1, which corresponds to a rather weak suppression.

To calculate the density of states in the S/F bilayer we should set the boundary conditions at the outer boundary of the ferromagnet (*x* = 0),

[9]
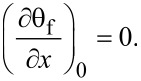


To complete the boundary problem we also set a boundary condition at *x* = ±∞,

[10]
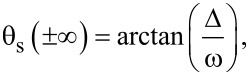


where the Green’s functions acquire the well-known bulk BCS form. We notice that the density of states at *x* = ±∞ is given by standard BCS equation,

[11]
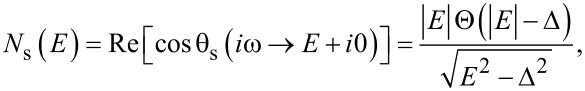


where Θ(*x*) is the Heaviside step function.

Finally the self-consistency equation for the superconducting order parameter takes the form,

[12]
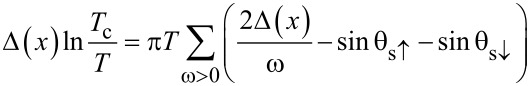


Equations Equation 5–Equation 10 and Equation 12 represent a closed set of equations that should be solved self-consistently.

The density of states *N*_f1,2_(*E*) normalized to the DOS in the normal state, can be written as

[13]
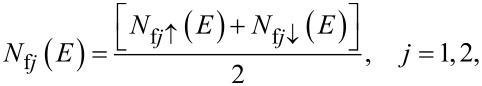


where *N*_f_*_j_*_↑(↓)_(*E*) are the spin-resolved densities of states written in terms of the spectral angle θ,

[14]
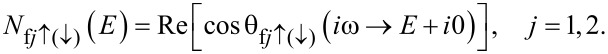


To obtain *N*_f1,2_, we use a self-consistent two-step iterative procedure [95,100–102]. In the first step we calculate the pair potential coordinate dependence Δ(*x*) using the self-consistency equation in the S layer (Equation 12). Then, by proceeding to the analytical continuation in Equation 5 and Equation 6 over the quasiparticle energy *i*ω→*E* + *i*0 and using the Δ(*x*) dependence obtained in the previous step, we find the Green’s functions by repeating the iterations until convergency is reached.

The characteristic lengths of the decay and oscillations of the real part of the pair wave function in the ferromagnetic layer at the Fermi energy, ξ_f1,2_, are given in our model by [45],

[1]
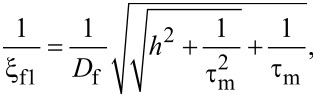


[2]
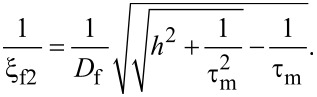


We see from these equations that with an increase of the magnetic scattering rate α_m_ = 1/τ_m_Δ the length of decay ξ_f1_ decreases, while the length of oscillations ξ_f2_ increases. In the absence of magnetic scattering ξ_f1_ = ξ_f2_ = ξ*_h_* = 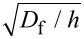.

## Results and Discussion

In this section we present the results of the DOS energy dependencies in SF bilayers at the free boundary of the F layer for *h* ≤ Δ. The densities of states for *h* ≥ Δ were thoroughly discussed in [45]. Then we calculate the corresponding CVC of the SFIFS junction using the Werthamer formula (Equation 3). In the case of *h* ≤ Δ we obtain an interesting nonmonotonic behavior of the quasiparticle current, presented in a subsection below (“Current–voltage characteristics of SFIFS junctions”). At large exchange fields the decay length ξ_f2_ of the real part of the pair wave function in the F layer becomes small (see Equation 1 and Equation 2), and the amplitude of DOS variations tends to zero. In this case the CVC of SFIFS junction tends to follow Ohm’s law for *h* ≫ Δ. The ferromagnetic materials with small exchange fields can be fabricated as discussed in [103]. We also note that the DOS at the end of an SF bilayer in case of a domain wall in the ferromagnetic layer was studied in [104].

### Density of states in SF bilayers for *h* ≤ Δ

**Figure 2:**
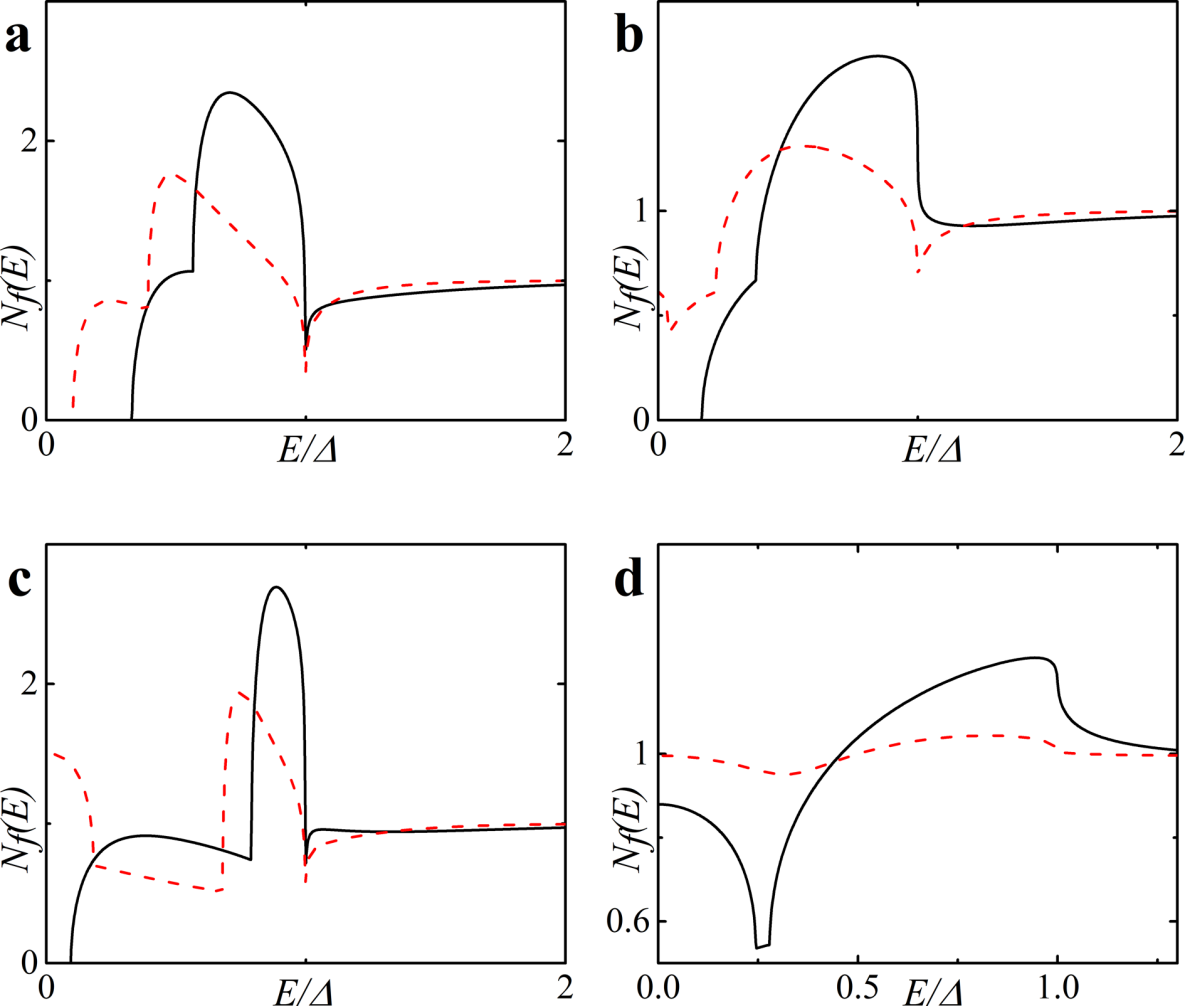
DOS *N*_f_(*E*) on the free boundary of the F layer in the FS bilayer obtained numerically for two cases: (a) in the absence of magnetic scattering, α_m_ = 1/τ_m_Δ = 0 (plots a and c) and in the case of finite magnetic scattering, i.e., plot b with α_m_ = 0.1 and plot d with α_m_ = 0.5. Parameters of the FS interface are γ = γ_B_ = 0.01, and *T* = 0.1*T*_c_. Plots a, b: *h* = 0.1Δ; plots c, d: *h* = 0.3Δ. The black solid line corresponds to *d*_f_ = 2ξ*_n_*, while the red dashed line corresponds to *d*_f_ = 3ξ*_n_*.

**Figure 3:**
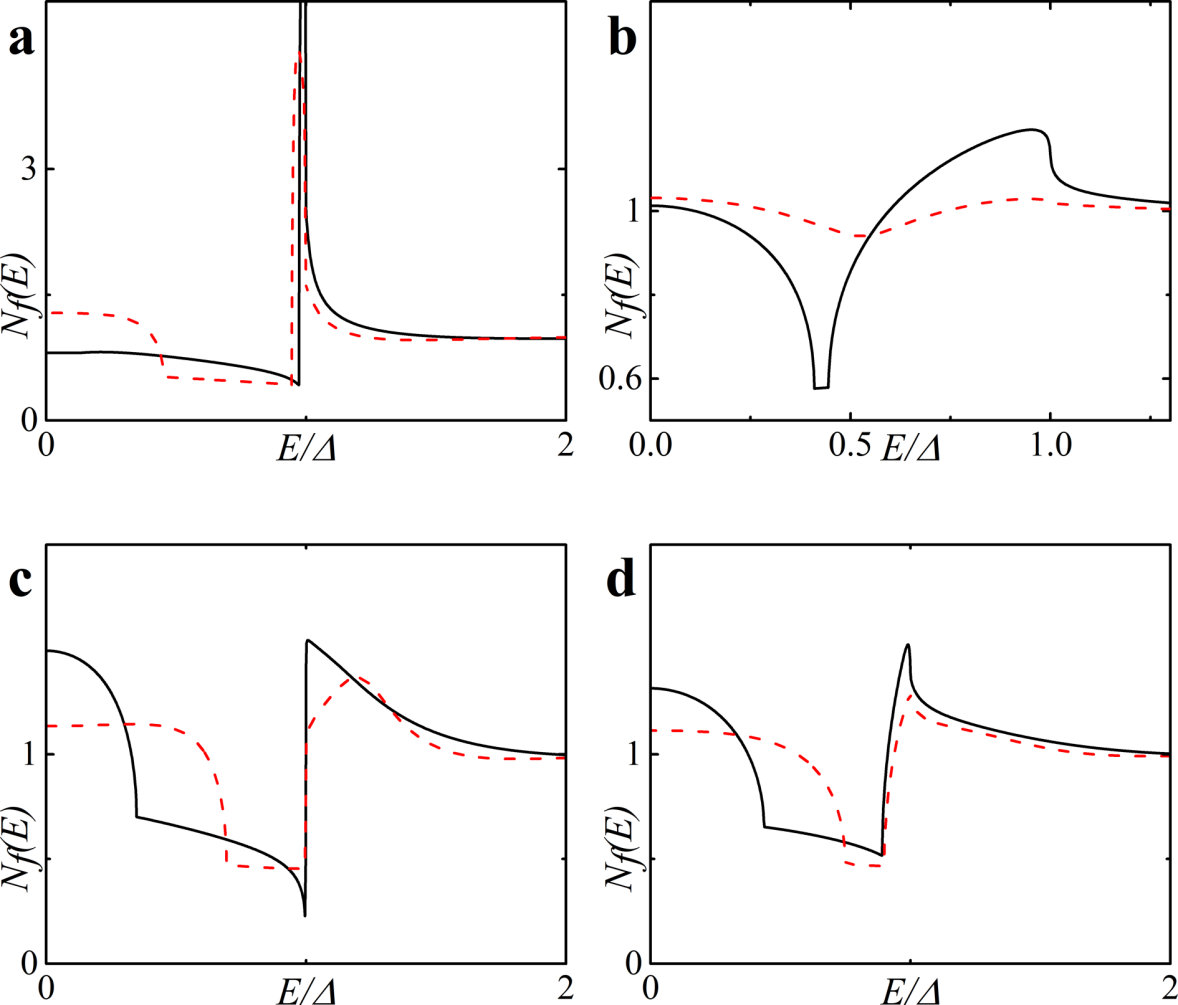
DOS *N*_f_(*E*) on the free boundary of the F layer in the FS bilayer obtained numerically in the absence of magnetic scattering, α_m_ = 1/τ_m_Δ = 0 (plots a and c) and in the case of finite magnetic scattering, i.e., plot b with α_m_ = 0.1 and plot d with α_m_ = 0.5. Plots a, b: *h* = 0.5Δ; plots c, d: *h* = 0.7Δ. The black solid line corresponds to *d*_f_ = 2ξ*_n_*, while the red dashed line corresponds to *d*_f_ = 3ξ*_n_*.

Figure 2 and Figure 3 show the DOS energy dependencies for different values of *h* ≤ Δ and for relatively thick F layers. In our calculations we fix the temperature at *T* = 0.1*T*_c_, where *T*_c_ is the critical temperature of the superconductor S. In Figure 2 the characteristic “finger-like” shape of DOS is observed along with a minigap for *d*_f_ = 2ξ*_n_* (Figure 2a,c). At larger *d*_f_ and/or at larger *h* the minigap closes (Figure 2c and Figure 3a,c)]. In the absence of magnetic scattering (α_m_ = 1/τ_m_Δ = 0) we can roughly estimate the critical value *h*_c_ of the exchange field at which the minigap closes as [45]

[15]
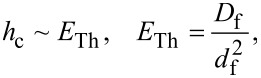


where *E*_Th_ is the Thouless energy and *d*_f_ is the thickness of the F layer in the SF bilayer (*d*_f1_ or *d*_f2_ for the left or right SF bilayer, respectively, in Figure 1). Since we consider subgap values of *h*, the minigap closes at rather large values of *d*_f_ in the absence of magnetic scattering.

After the minigap closes the DOS at the Fermi energy *N*_f_(0) rapidly increases to values larger than unity with further increase of *d*_f_ and then it oscillates around unity while its absolute value exponentially approaches unity [45]. This is the well-known damped oscillatory behavior with the lengths of decay and oscillations given by Equation 1 and Equation 2, respectively. Figure 2b,d and Figure 3b,d show that stronger magnetic scattering leads to the minigap closing at smaller values of *d*_f_. With the increase of α_m_ = 1/τ_m_Δ the period of oscillations increases (ξ_f2_ in Equation 2 increases). At the same time the DOS variation amplitude becomes smaller and DOS features smear, since for larger α_m_ the dumped exponential decay of oscillations occurs faster (ξ_f1_ in Equation 1 decreases).

**Figure 4:**
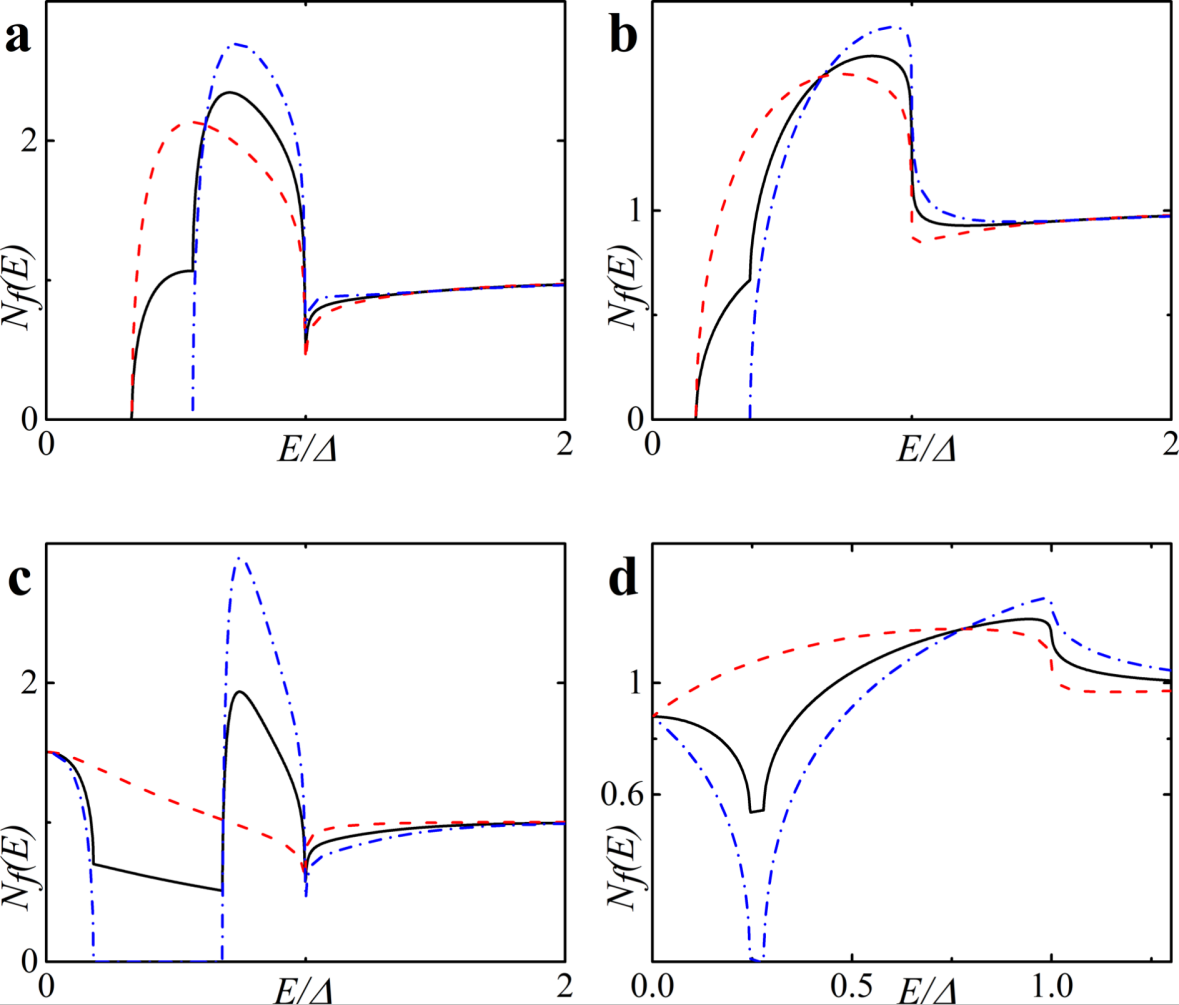
Spin-resolved DOS *N*_f↑(↓)_ on the free boundary of the F layer in the FS bilayer obtained numerically in the absence of magnetic scattering, α_m_ = 0 (plots a and c) and in the case of finite magnetic scattering, i.e., plot b with α_m_ = 0.1 and plot d with α_m_ = 0.5. Plots a, b: *h* = 0.5Δ, *d*_f_ = 2ξ*_n_*; plots c, d: *h* = 0.3Δ, *d*_f_ = 3ξ*_n_* (c) and *d*_f_ = 2ξ*_n_* (d). The black solid line corresponds to *N*_f_(*E*), the red dashed line corresponds to *N*_f↑_(*E*) and the blue dash-dotted line corresponds to *N*_f↓_(*E*).

Finally, in Figure 4 we present plots for spin-resolved densities of states given by Equation 14 for both zero and finite magnetic scattering.

### Current–voltage characteristics of SFIFS junctions

Using the densities of states *N*_f1,2_(*E*) obtained in the subsection above, we calculate a set of quasiparticle current curves using Equation 3 for various values of parameters describing properties of ferromagnetic material, which include the thicknesses of the F layers, *d*_f1_ and *d*_f2_, the exchange field *h*, and the magnetic scattering rate α_m_. In our calculations we fix the temperature at *T* = 0.1*T*_c_, where *T*_c_ is the critical temperature of the superconducting lead.

**Figure 5:**
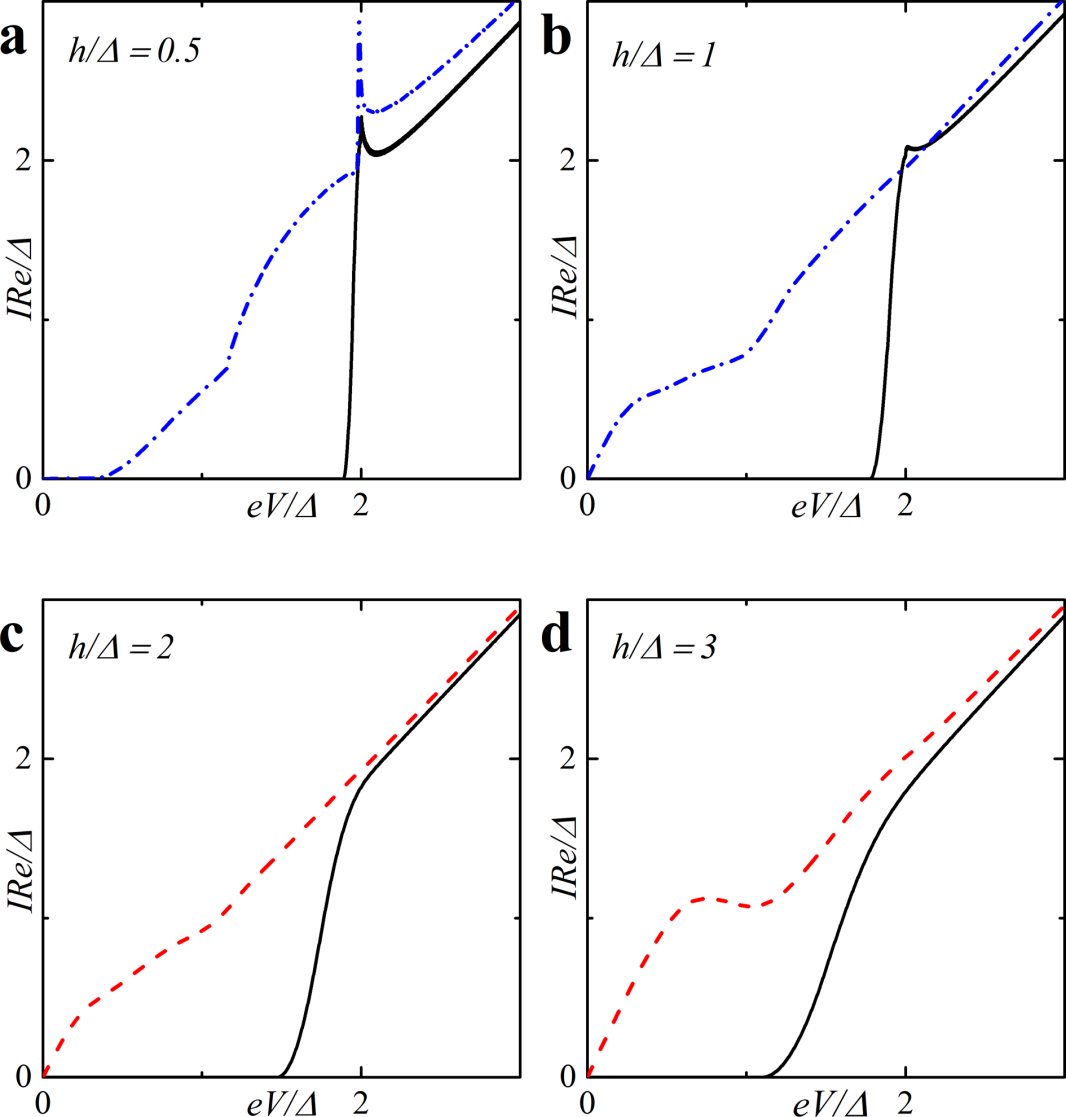
Current–voltage characteristics of the symmetric (*d*_f1_ = *d*_f2_ = *d*_f_) SFIFS junction in the absence of magnetic scattering for different values of exchange field *h*. The temperature *T* = 0.1*T*_c_. In each graph the curves were calculated for different values of F layer thickness *d*_f_, *d*_f_ = 0.5ξ*_n_* (black solid line), *d*_f_ = 1.0ξ*_n_* (red dashed line), *d*_f_ = 1.5ξ*_n_* (blue dash-dotted line). The plots correspond to specific values of the exchange field *h* : plot (a) to *h* = 0.5Δ, (b) to *h* = 1.0Δ, (c) to *h* = 2.0Δ and (d) to *h*= 3.0Δ.

Figure 5 shows the CVC of a symmetric SFIFS junction, where *d*_f1_ = *d*_f2_ = *d*_f_ in the absence of magnetic scattering. For thin enough ferromagnetic interlayers, *d*_f_/ξ*_n_* = 0.5, and a small enough value of the exchange field, *h* = 0.5Δ, we observe CVC that resemble the *I*–*V* characteristic of a SNINS Josephson junction with a characteristic peak at *eV* ≈ 2Δ (see Figure 5a, solid black line) [101]. With an increase of the exchange field *h* this peak becomes smeared (see Figure 5b–d, solid black line). Increasing *d*_f_ and/or *h* produces a set of *I*–*V* curves among which the red dashed line in Figure 5d is the most interesting because it exhibits a nonmonotonic behavior. The reason of this atypical nonmonotonic behavior will be explained later.

**Figure 6:**
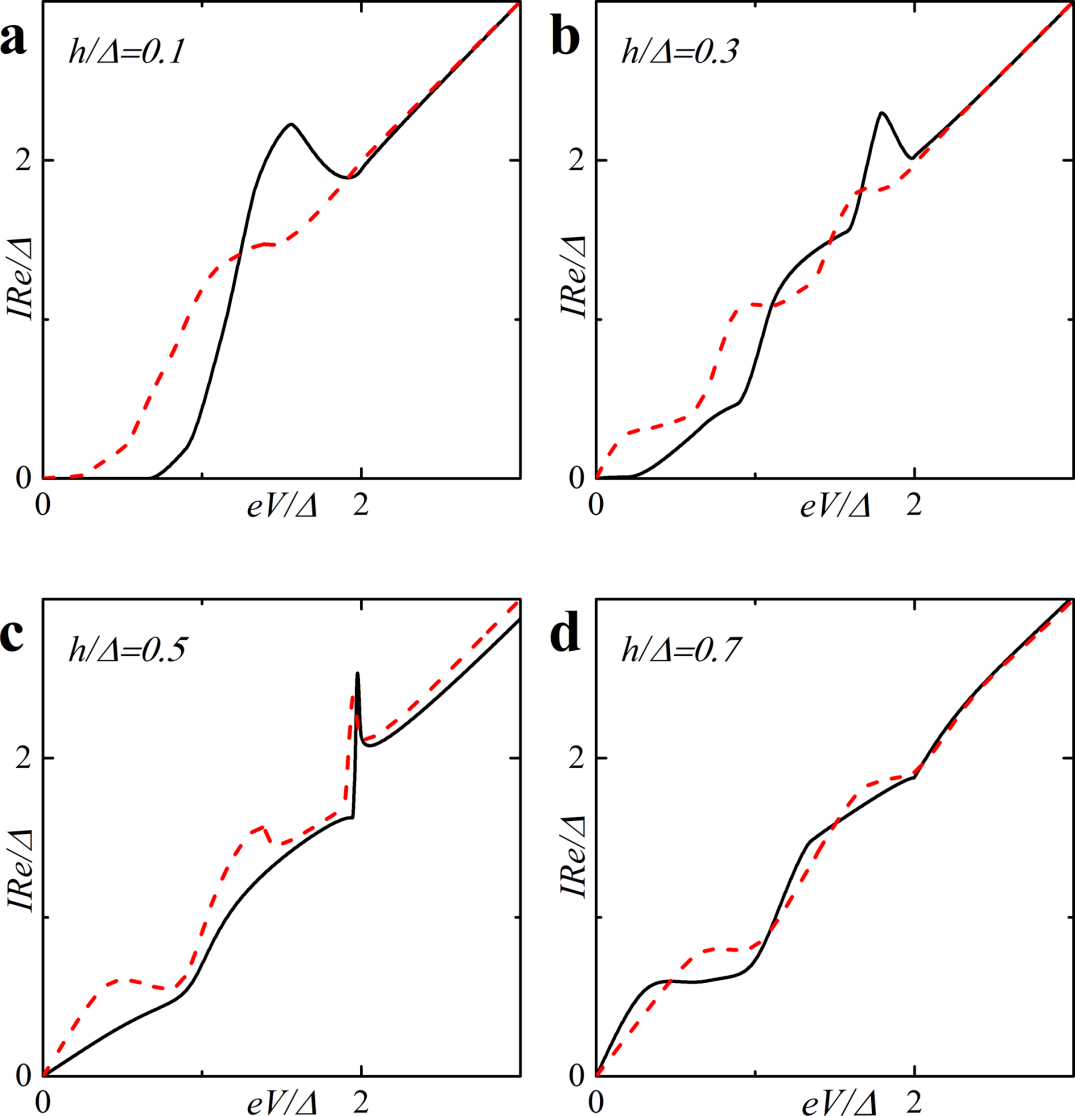
Current–voltage characteristics of a symmetric SFIFS junction for different values of the subgap exchange field *h* in the absence of magnetic scattering at a temperature *T* = 0.1*T*_c_. In each graph the curves were calculated for different values of F layer thickness, *d*_f_, *d*_f_ = 2ξ*_n_* (black solid line) and *d*_f_ = 3ξ*_n_* (red dashed line). The plots correspond to specific values of the subgap exchange field *h*: plot (a) to *h* = 0.1Δ, (b) to *h* = 0.3Δ, (c) to *h* = 0.5Δ and (d) to *h* = 0.7Δ.

**Figure 7:**
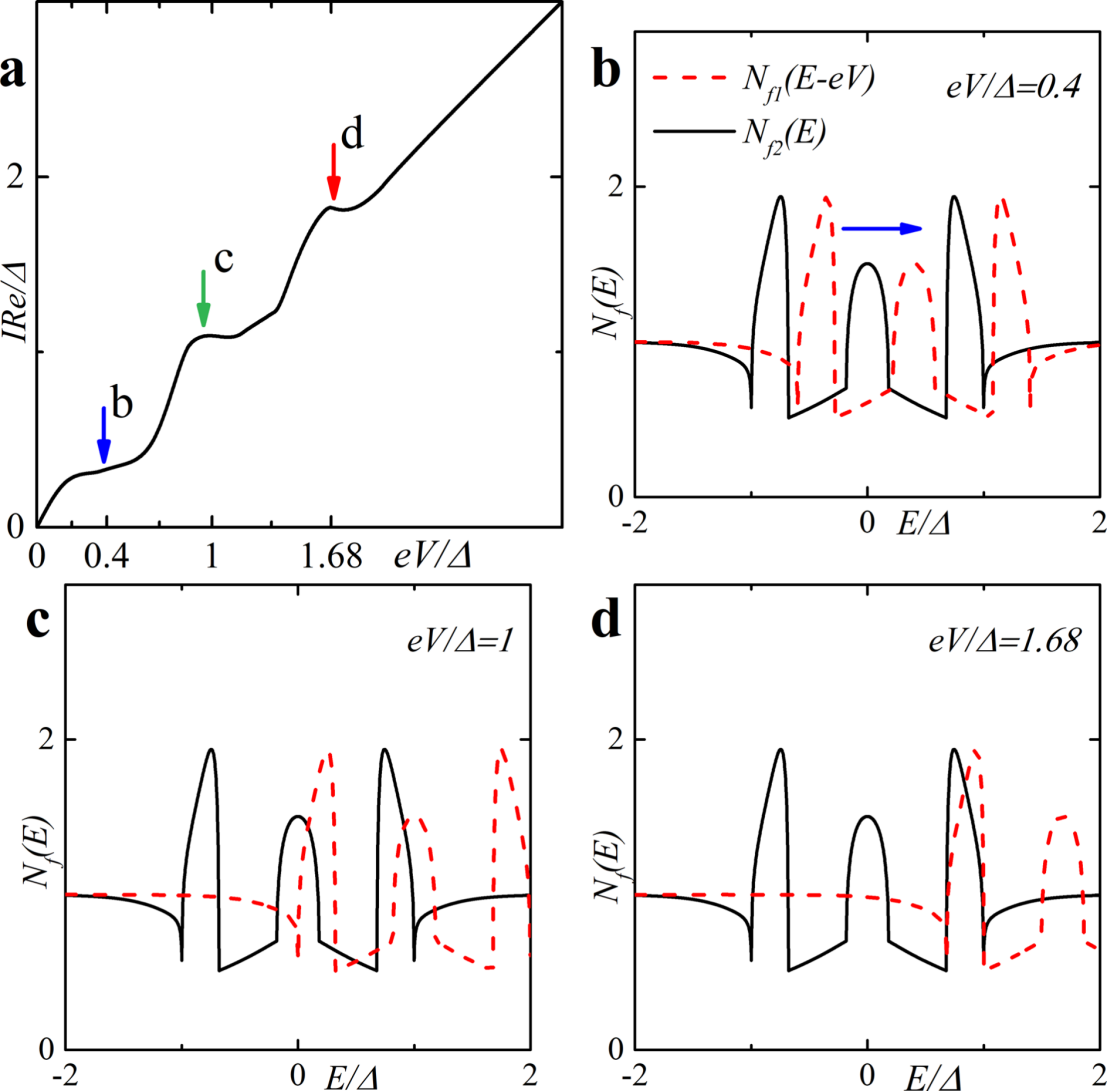
(a) CVC taken from Figure 6b, red dashed line, and visual explanation of the characteristic behavior of the quasiparticle current. (b–d) The DOS *N*_f_(*E* − *eV*) and *N*_f_(*E*) at particular values of *eV* revealing the origin of the current features in plot (a).

Figure 6 shows the current–voltage characteristics of SFIFS junctions at subgap values of the exchange field. We observe a nonmonotonic behavior for thick enough ferromagnetic layers at *h* ≤ Δ. Let us consider the CVC in Figure 6b, red dashed line. We can explain its behavior as well as any other nonmonotonic CVC behavior as the signature of the DOS energy dependence. The anomalous nonmonotonic *I*(*V*) dependence arises from the shape features of the densities of states, see Figure 7. In symmetric SFIFS junctions, *N*_f1_(*E*) = *N*_f2_(*E*) ≡ *N*_f_(*E*) in Equation 3, which can be well approximated by taking *T* = 0 for small temperatures *T* ≪ *T*_c_. In this case the Fermi–Dirac distribution function *f*(*E*) can be represented with the Heaviside step function Θ(*−E*) [and *f*(*E* − *eV*) with Θ(*eV* − *E*)]. As a result, the limits of integration in Equation 3 shrink to the interval [0, *eV*]. Hence, the current through the junction can be written as,

[16]
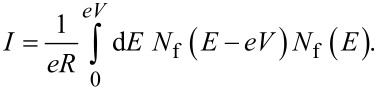


Using this expression, the origin of the nonmonotonic behavior of the CVC can be explained. At *eV* = 0 the upper limit of the integral in Equation 16 is zero and the current is zero. With the increase of the voltage, the current first increases linearly due to the broader region of integration as in Ohm’s law. The first feature that is shown in Figure 7a is a significant change in the slope of the current. Figure 7b shows the relative positions of the densities of states *N*_f_(*E* − *eV*) and *N*_f_(*E*) in this case, where almost no peak overlap can be seen, resulting in relatively small values of the integral in Equation 16. As we proceed to larger values of *eV*, we reach the first local maximum of the CVC, which corresponds to a maximum overlap of the densities of states *N*_f_(*E* − *eV*) and *N*_f_(*E*) at *eV*/Δ ≈ 1 (see Figure 7c). The second maximum of the quasiparticle current occurs at *eV*/Δ ≈ 1.68, which corresponds to a perfect DOS peak overlap at *E*/Δ ≈ 1 Figure 7d). For large enough values of the voltage *eV*, a product of the DOS *N*_f_(*E* − *eV*) *N*_f_(*E*) ≈ 1 and its integration does not produce any features. Thus, the CVC eventually coincides with Ohm’s law in this case. In fact any shape of a SFIFS *I*–*V* curve can be explained and understood in this way. We note that in this paper we present the densities of states in SF bilayers only for subgap values of the exchange field. For *h* ≥ Δ the DOS energy dependencies in SF bilayers can be found in [45].

**Figure 8:**
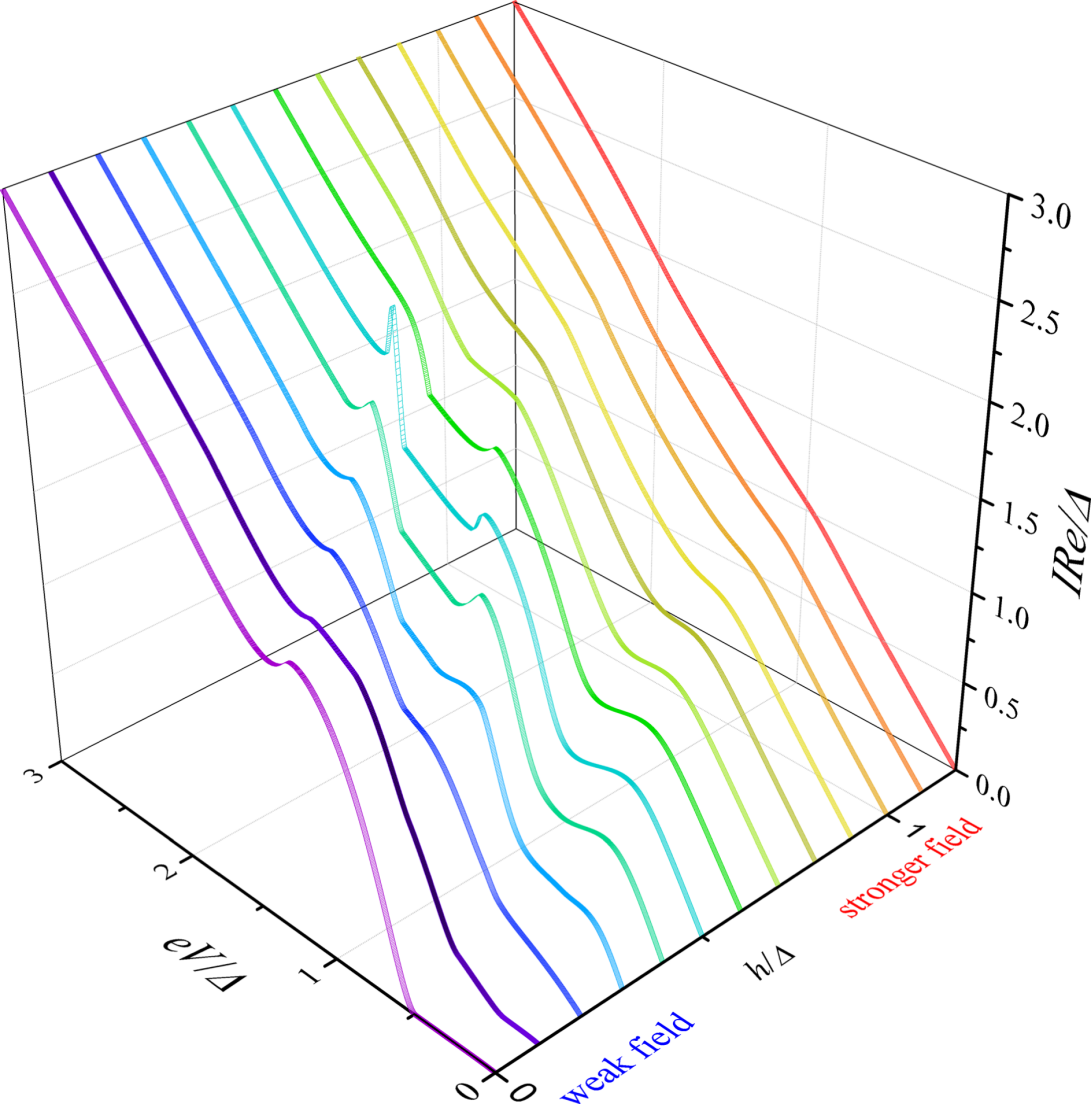
Current–voltage characteristics of a symmetric SFIFS junction in the absence of magnetic scattering for *d*_f_ = 3ξ*_n_*. The temperature is *T* = 0.1*T*_c_. The curves correspond to different values of *h*, from *h* = 0 to *h* = 1.2Δ with increments equal to 0.1Δ. The exchange field *h* = 0 corresponds to the case of a SNINS junction [101].

**Figure 9:**
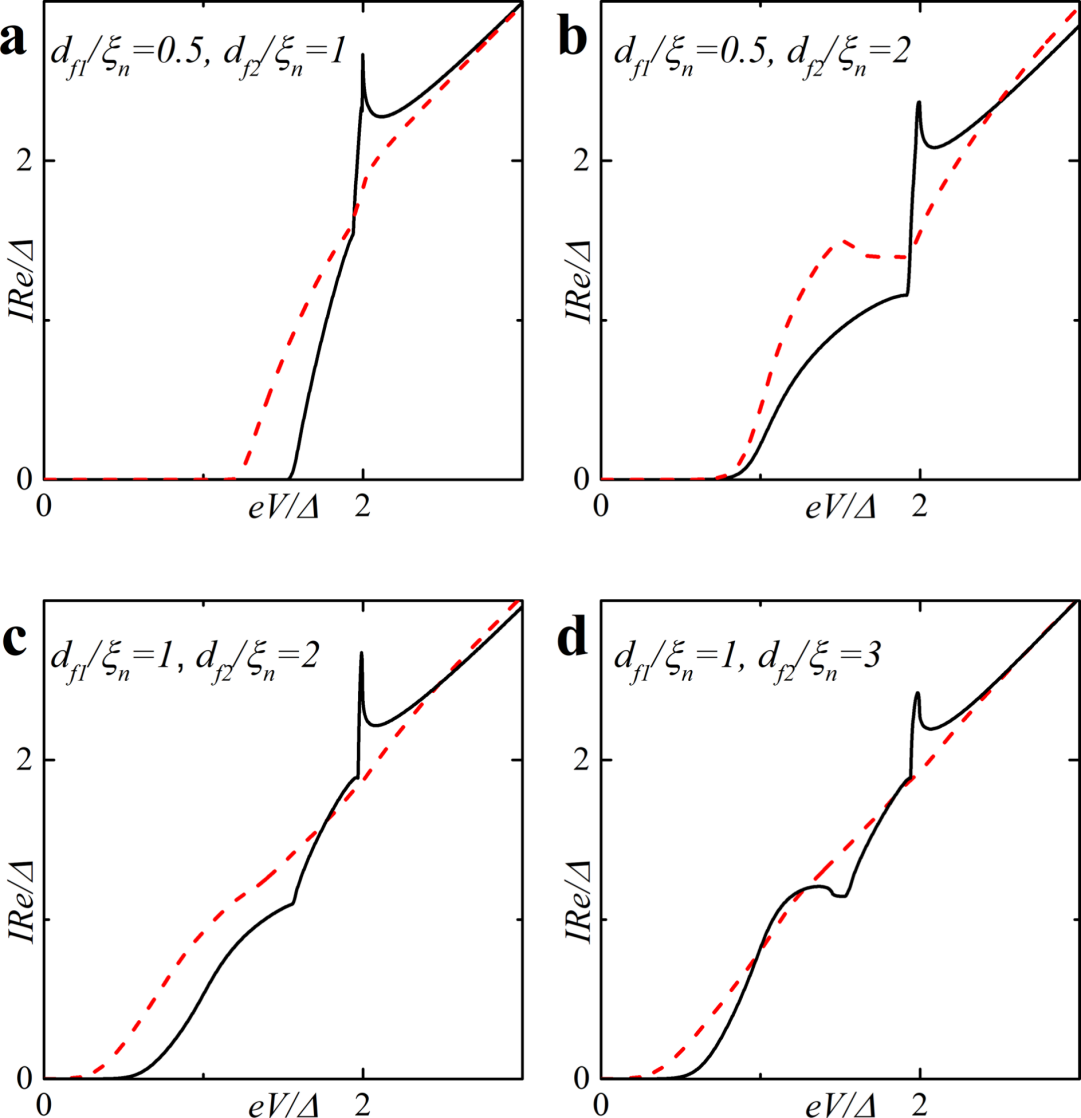
Current–voltage characteristics of an asymmetric (*d*_f1_ ≠ *d*_f2_) SFIFS junction for different values of F layer thicknesses *d*_f1_ and *d*_f2_ (indicated in the plot) in the absence of magnetic scattering. The temperature is *T* = 0.1*T*_c_, *h* = 0.5Δ (black solid line) and *h* = 1.0Δ (red dashed line). The labels show the values of F layer thicknesses *d*_f1_ and *d*_f2_ for which the curves were calculated: plot (a) corresponds to *d*_f1_ = 0.5ξ*_n_* and *d*_f2_ = 1.0ξ*_n_*, (b) to *d*_f1_ = 0.5ξ*_n_* and *d*_f2_ = 2.0ξ*_n_*, (c) to *d*_f1_ = 1.0ξ*_n_* and *d*_f2_= 2.0ξ*_n_* and (d) to *d*_f1_ = 1.0ξ*_n_* and *d*_f2_ = 3.0ξ*_n_*.

Based on the properties of the density of states in FS bilayers we can see that even the tiny exchange field *h* can dramatically modify the current introducing anomalous nonmonotonic behavior in case of thick enough F layers (see Figure 5 and Figure 6). It is important to understand how the CVC of a SFIFS junction transforms as the exchange field *h* increases. In Figure 8 we demonstrate the plot of current–voltage characteristics calculated for a wide range of exchange field values *h* in the absence of magnetic scattering. From this plot it can be clearly seen that while for relatively small (subgap) values of the exchange field many interesting features appear in the structure of the current, at larger values of *h* these features are smeared and the CVC approaches Ohm’s law. Figure 9 shows the current–voltage characteristics in the case of an asymmetric SFIFS junction, i.e., when *d*_f1_ ≠ *d*_f2_ in the scase of zero magnetic scattering.

**Figure 10:**
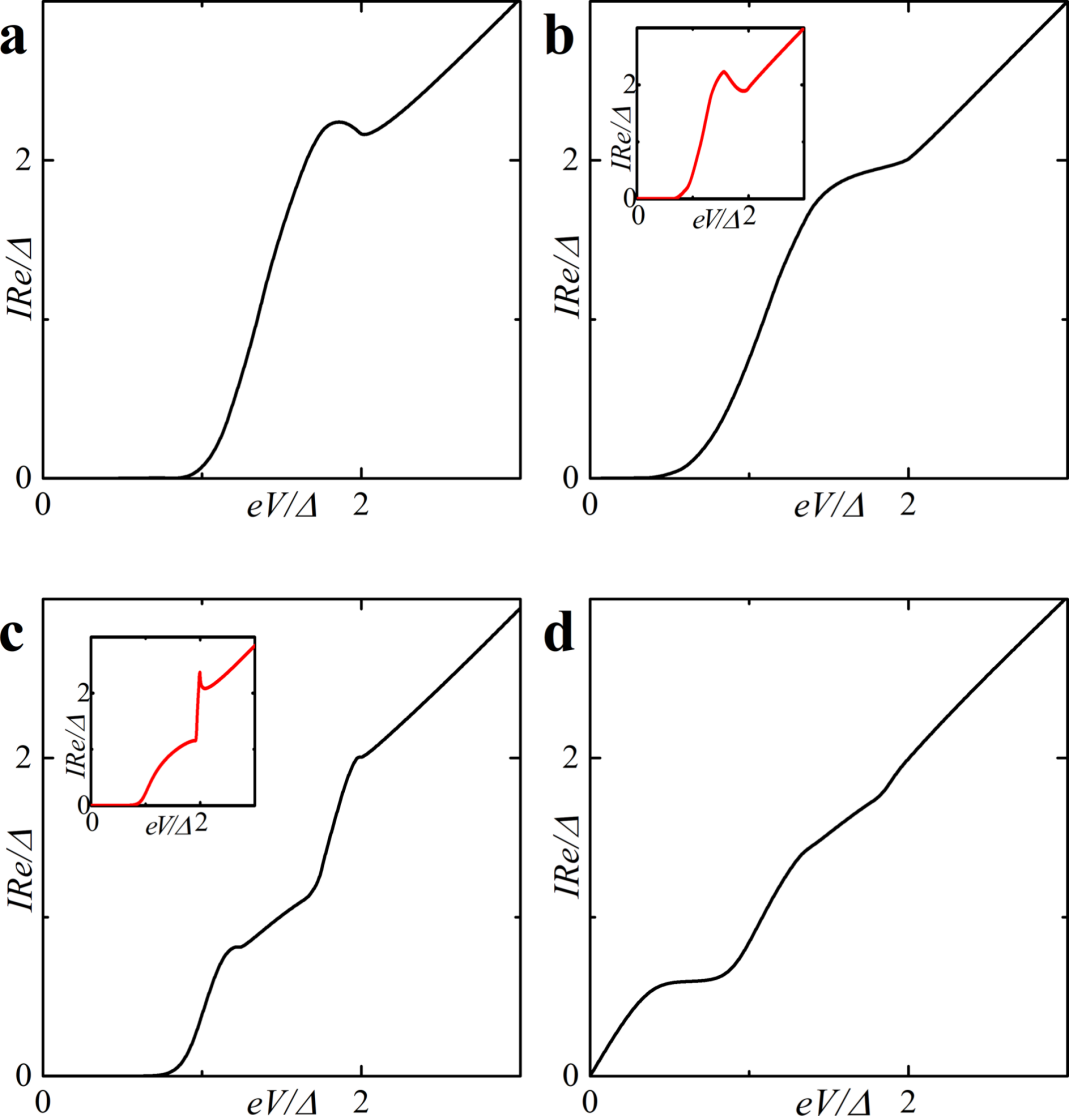
Current–voltage characteristics of a SFIFS junction in the presence of magnetic scattering (α_m_ = 0.1). The temperature is *T* = 0.1*T*_c_. In plot (a) the black solid line corresponds to *d*_f1_ = 1ξ*_n_*, *d*_f2_ = 2ξ*_n_*, in plots (b) and (d) to *d*_f1_ = *d*_f2_ = 2ξ*_n_*, and finally in plot (c) the black line corresponds to *d*_f1_ = 0.5ξ*_n_*, *d*_f2_ = 2ξ*_n_*. Plots (a, b): *h* = 0.1Δ; plot (c) and plot (d): *h* = 0.5Δ and *h* = 0.7Δ, respectively. The insets show the CVC in case of zero magnetic scattering.

In this section we also present the current–voltage characteristics of a SFIFS junction calculated in the presence of magnetic scattering for different values of the subgap exchange field *h*. Figure 10 illustrates the CVC in case of a finite magnetic scattering rate α_m_ = 0.1. We consider both symmetric and asymmetric SFIFS junctions. The insets show the CVC in case of zero magnetic scattering. For very small *h* nonzero magnetic scattering leads to smearing of characteristic features of the current as shown in Figure 10. At larger subgap values of the exchange field *h* we see a “triple kink” structure (Figure 10c). For large enough values of α_m_ the nonmonotonic behavior of the quasiparticle current will be smeared and the current approaches Ohm’s law. This is due to the fact that increasing α_m_ the length of the superconducting correlations decay in the ferromagnetic layers decreases, see Equation 1, and the suppression of superconducting correlations in the F layers occurs faster.

We can compare these results with the *I*–*V* characteristics of SIFS Josephson junctions [45]. In this case at zero magnetic scattering we may also observe the nonmonotonic behavior, but with only one peak [see Ref. [45], Figure 6 (c)]. In case of finite magnetic scattering the CVC has a “double kink” structure [see Ref. [45], Figure 7 (a, c)]. In SFIFS junctions the overlap of subgap DOS structures *N*_f1_(*E* − *eV*) *N*_f2_(*E*) in the integrand of the current equation, Equation 16, produce more complex behavior of the *I*–*V* characteristics.

We also notice that in recent experiments on SFIFS junctions as injectors of superconductor-ferromagnetic transistors some fine structures of the subgap quasiparticle current was observed [82–85], which looks similar to our theoretical results.

## Conclusion

In this work we have presented the results of CVC calculations of a SFIFS junction for different set of parameters including the thicknesses of the ferromagnetic layers, *d*_f1_ and *d*_f2_, the exchange field, and the magnetic scattering time α_m_ = 1/τ_m_Δ. We considered the case of a strong insulating barrier such that the left SF and the right FS bilayers are decoupled. In order to obtain the current–voltage characteristics we first calculated the densities of states on the free boundary of the F layer in each SF bilayer utilizing an iterative self-consistent approach. Using the numerically obtained DOS we have derived the quasiparticle current of a SFIFS junction in the case of symmetric (*d*_f1_ = *d*_f2_) and asymmetric (*d*_f1_ ≠ *d*_f2_) structures. We have paid much attention to the case of a SFIFS junction with weak ferromagnetic interlayers with exchange fields *h* ≤ Δ. It was demonstrated that the CVC exhibits interesting and unusual features in this case, which can be ascribed to typical DOS behavior. We have provided a simple physical explanation for such anomalous CVC behavior. We have also illustrated how the CVC shape evolves as one increases the exchange field *h*. It should be emphasized that taking into account finite magnetic scattering leads to the smearing of characteristic features and, in particular cases, to a “triple kink” shape of the current.
